# Fine-scale sampling unveils diazotroph patchiness in the South Pacific Ocean

**DOI:** 10.1038/s43705-021-00006-2

**Published:** 2021-03-25

**Authors:** Mar Benavides, Louis Conradt, Sophie Bonnet, Ilana Berman-Frank, Stéphanie Barrillon, Anne Petrenko, Andrea Doglioli

**Affiliations:** 1 Aix Marseille Univ, Université de Toulon, CNRS, IRD, MIO UM 110, Marseille, France; 2 Department of Marine Biology, Leon H. Charney School of Marine Sciences, University of Haifa, Mt. Carmel, Haifa, Israel

**Keywords:** Biogeography, Biogeochemistry

## Abstract

Diazotrophs are important contributors to nitrogen availability in the ocean. Oceanographic cruise data accumulated over the past three decades has revealed a heterogeneous distribution of diazotroph species at regional to global scales. However, dynamic fine-scale physical structures likely affect the distribution of diazotrophs at smaller spatiotemporal scales. The interaction between fine-scale ocean dynamics and diazotrophs remains poorly understood due to typically insufficient spatiotemporal sampling resolution and the lack of parallel detailed physical studies. Here we show the distribution of five groups of diazotrophs in the South Pacific at an unprecedented resolution of 7–16 km. We find a patchy distribution of diazotrophs, with each group being differentially affected by parameters describing fine-scale physical structures. The observed variability in species abundance and distribution would be masked by a coarser sampling resolution, highlighting the need to consider fine-scale physics to resolve the distribution of diazotrophs in the ocean.

The surface ocean is constantly stirred by currents that swirl and mix different seawater masses creating a dynamic mosaic of biogeochemical properties.^1^ Numerical modeling and satellite data show that “fine-scale” structures such as filaments and eddies (with typical spatiotemporal scales of 1–100 km and days-weeks) impact the distribution of phytoplankton and carbon export in the ocean.^2–4^ While these remote approaches provide synoptic views at large spatial scales, at-sea sampling remains imperative to resolve the diversity, metabolism and trophic interactions of microbes at finer scales. Yet, the typical spatiotemporal sampling resolution is too coarse to resolve fine-scale processes.^5^

Understanding the effect of fine-scales on biogeochemically relevant microbes is of particular importance. Dinitrogen (N_2_) fixers or “diazotrophs” provide a significant source of bioavailable nitrogen to the ocean.^6^ Diazotrophs may accumulate in anticyclonic eddies,^7,8^ where eddy pumping deepens isopycnals impoverishing surface waters in inorganic nitrogen presumably favoring diazotroph growth.^9^ However, diazotrophs also accumulate in cyclonic eddies where low nutrient concentrations are attributed to wind-driven Ekman pumping.^10^ Such complex interactions between fine-scale physics and diazotrophs cannot be understood from satellite data alone.

Diazotrophs have a variable tolerance to biogeochemical conditions. For example, photosynthetic cyanobacteria such as *Trichodesmium* and UCYN-B abound in oligotrophic (sub)tropical waters, while UCYN-A’s distribution spans the tropics to polar seas.^6^ Non-cyanobacterial diazotrophs cannot photosynthesize, and thus are regulated by different drivers to obtain carbon and energy.^11^ With such divergent physiologies, it is unlikely that different diazotrophs respond to fine-scale forcing similarly. Resolving these ambiguities requires coupling fine-scale physical measurements and diazotroph activity/abundance data at high spatiotemporal resolution. The vast majority of published diazotroph data were obtained at locations ~160 km apart (median distance between stations in the diazotroph database^12^). Robidart et al.^13^ quantified diazotrophs with an ecogenomic sensor drifting over a single eddy at a resolution of ~30 km. More recently, Tang et al.^14^ showed underway diazotroph activity/abundance data with ~18 km resolution, but the effect of fine-scale physics was not taken into account.

Here we demonstrate the fine-scale distribution of diazotrophs in three zones of intense fine-scale activity in the South Pacific (TONGA cruise doi: 10.17600/18000884; Fig. 1). Each zone was selected for high-resolution sampling according to satellite and Lagrangian product maps received onboard on a daily basis.^15^ Maps included absolute dynamic topography (ADT), used to compute geostrophic velocities; finite-size Lyapunov exponents (FSLE), which depict transport barriers created by currents that can represent ecological boundaries; and the Okubo-Weiss parameter (OW), which distinguishes vorticity-dominated from strain-dominated zones (e.g., eddies vs. non-eddies) (Supplementary Information; Table S1; Fig. S1). These parameters are thus useful to quantify fine-scale structures and to study their covariability with plankton distribution [e.g., ^4^]. Planktonic biomass was collected with an automated filtration system at an unprecedented resolution of 7–16 km. DNA extracted from the filters was used to quantify five diazotroph groups (*Trichodesmium*, UCYN-A1, UCYN-B, UCYN-C, and Gamma A) by quantitative PCR targeting the *nifH* gene, and, only in zone 3 nutrient concentrations and N_2_ fixation rates were also measured (Supplementary Information).

**Fig. 1:**
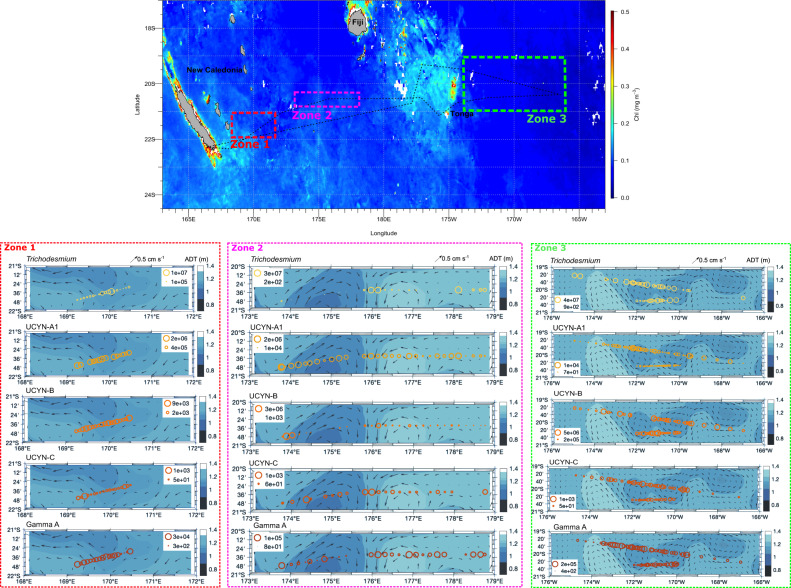
Fine-scale resolution abundance of diazotrophs along three selected sampling zones. The top image shows a chlorophyll MODIS composite averaged for November 2019 at a resolution of 4 km and the location of the three selected sampling zones. The three lower panels (in dotted squares) show the abundance of diazotrophs (*Trichodesmium*, UCYN-A1, UCYN-B, UCYN-C, and Gamma) in each selected zone as *nifH* gene copies per liter of seawater. Diazotroph abundances (*nifH* gene copies l^−1^) are superimposed on absolute dynamic topography (ADT, color scale) and geostrophic velocity (arrows). ADT data were retrieved for each zone on 2nd, 4th and 22nd November 2019 for zones 1, 2, and 3, respectively.

Our results reveal a patchy distribution of diazotrophs, driven by a heterogeneous effect of fine-scale physical parameters on each group (Fig. 1; Fig. S2; Table S2). *Trichodesmium* correlated positively with ADT (Fig. 1; Fig. S2; Table S2) and accumulated at positive-negative OW transition and high FSLE regions located at ~170 °E, 176 °E, and 171 °W in zones 1, 2 and 3, respectively; Fig. 2). A particularly striking accumulation of *Trichodesmium* was observed at the convergence of two counter-rotating eddies in zone 2 (maximum 3 × 10^7^ gene copies l^−1^; Fig. 1), coinciding with high FSLE values (Fig. 2) and a steep change in temperature (Fig. S3). The concentration of *Trichodesmium* at fronts depicted by high FSLE is likely linked to intracellular gas-vesicles providing them positive buoyancy.^16^ Previous concentrations of *Trichodesmium* along FSLE ridges uncoupled from significant N_2_ fixation rates have been interpreted as “passive” accumulations, i.e. fine-scale dynamics affecting their distribution but not their activity.^17^

**Fig. 2:**
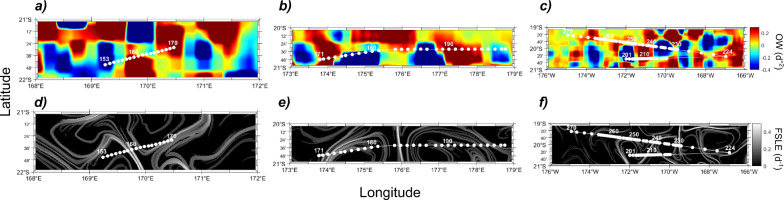
Lagrangian diagnostics parameters. **a**–**c** show values of the Okubo-Weiss (OW) in zones 1, 2, and 3, respectively. **d**–**f** show values of finite Lyapunov finite size Lyapunov exponents (FSLE) in zones 1, 2, and 3, respectively. White dots represent sampling locations, station numbers are also shown in white font.

UCYN-A1 were negatively related to FSLE (Table S2) and to *Trichodesmium* (Figs. S2 and S4), in agreement with the antagonistic biogeographic trends of these two diazotrophs witnessed at larger spatial scales.^12^ In comparison to other groups, the distribution of UCYN-A1 and UCYN-B was more spatially homogeneous and independent of fine-scales (Fig. S2). They were instead positively correlated to phosphate concentrations (Fig. S5), suggesting an “active” response to nutrient inputs induced by fine-scale dynamics^9^). These unicellular groups have a large surface area:volume ratio allowing for efficient nutrient utilization and higher growth rates than *Trichodesmium*,^6^ which could explain their faster response to limiting nutrient inputs induced by fine-scale dynamics. UCYN-C were significantly related to both ADT and FSLE (Table S2), but were the least abundant group (Fig. 1), likely due to their presumed coastal origin.^6^ Finally, Gamma A were significantly related to ADT (Table S2) and accumulated in frontal zones with high FSLE (Fig. 1), which agrees with their apparent particle-attached lifestyle.^18^

Although relatively high concentrations of phosphate were measured east of ~175°W (Fig. S6), they sustained only moderate N_2_ fixation rates (1–5 nmol N l^−1^ d^−1^; Fig. S6) likely due to a scarcity of iron east of the Tonga volcanic arc.^19^ N_2_ fixation rates correlated positively with *Trichodesmium* and negatively with UCYN-B (Fig. S5). Temperature and nutrients are typically invoked to define diazotroph biogeography on regional/global scales.^12^ These factors were remarkably homogeneously distributed in zone 3 (Figs. S6–7), and did not vary significantly according to fine-scale parameters (Fig. S7). Yet, diazotroph abundance revealed high spatial variability at the fine-scale in zone 3 (Fig. 1). Such variability would have gone unseen at a coarser resolution, stressing the role of fine-scale dynamics in diazotroph distribution.

The patchiness observed likely responds to a combination of bottom-up and top-down interactions between diazotrophs’ competitors, predators and their biogeochemical environment. Understanding the controls imposed by fine-scales on other biotic and abiotic drivers will enhance our understanding of current and future diazotroph distribution and role in supplying new nitrogen to the ocean.

## Supplementary information

Supplementary information
