# Supplementary material for: Fine-scale sampling unveils diazotroph patchiness in the South Pacific Ocean
Source: ISME Commun. 2021 Mar 25;1:3. doi: 10.1038/s43705-021-00006-2 (PMC9723698; doi:10.1038/s43705-021-00006-2)
Supplement: Supplementary file 1 — Supplementary information [file 43705_2021_6_MOESM1_ESM.docx]

**Supplementary Information**

**Table of contents**

1. Supplementary Methods

2. Supplementary Tables

3. Supplementary Figures

4. Supplementary References

**1. Supplementary Methods**

*Fine-scale features and high-resolution biomass sampling*

The TONGA cruise (“shallow hydroThermal sOurces of trace elements: potential impacts on biological productivity and the bioloGicAl carbon pump”; http://tonga-project.org, doi: 10.17600/18000884) took place onboard the *R/V L’Atalante* between 31^st^ October and 6^th^ December 2019. High-resolution sampling while transiting between stations targeted fine-scale structures shown in satellite product reports sent by the land team to embarked scientists on a daily basis. The daily report on fine-scale variability was processed with the software ‘Satellite-based Sampling for Oceanographic cruises’ (SPASSO: 'Software Package for an Adaptive Satellite-based Sampling for Oceanographic cruises' <https://spasso.mio.osupytheas.fr/>; [1]), which we have previously used to study the fine-scale distribution of different planktonic groups in the Mediterranean Sea, Southern and South Pacific Oceans [2–5]. SPASSO reports included images of sea surface temperature (SST), chlorophyll and geostrophic current based on absolute dynamic topography (ADT), obtained from the Copernicus Marine Environment Monitoring Service (<https://marine.copernicus.eu/>). The SPASSO products provided also included finite size Lyapunov exponents (FSLE) obtained by integrating the trajectories of several thousand numerical particles over 30 days, and the daily values of the Okubo-Weiss parameter (OW). FSLE values indicate the exponential rate of particle trajectory separation, allowing to identify Lagrangian Coherent Structures (LCS) from altimetry data as candidates for fine-scale frontal regions [6]. The OW parameter compares the importance of deformation of the current velocity field with respect to its rotation. Negative values of OW quantify the strength of an eddy, its velocity field being dominated by rotation.

SPASSO images were used to select three zones of relevant fine-scale activity for high-resolution sampling. Zones 1 and 2 ranged between 169.24°E - 170.46°E and between 173.78°E - 178.75°E, respectively. Zone 3 included one west-to-east (southern) and one east-to-west (norther) transect, which ranged between 171.99°W - 169.95°W and 166.98°W - 174.92°W, respectively. Zone 1 was sampled on 2^nd^ November and included 18 stations separated by an average distance of 7 km. Zone 2 was sampled between 4^th^ and 5^th^ November and included 30 stations with an average separation of 14 km. Finally, zone 3 included 23 stations on the forward transect sampled on 17^th^ November with an average separation of 7 km, while the return transect included 47 stations separated by 16.6 km on average.

In each zone seawater was pumped from 5 m depth through the ship’s underway outlet, which was washed with concentrated bleach and flushed with demineralized water for several hours before the cruise. Temperature and salinity were measured with a SBE21 thermosalinograph (Sea-Bird Scientific, Bellevue, WA, USA) and binned at 1 min. Fluorescence data were recorded every 10-15 min with a Wetstar fluorimeter (Sea-Bird Scientific). Plankton biomass was collected by an OCE-5 autosampler (Oceomics, Fuerteventura, Spain) connected to the ship’s underway. Briefly, the OCE-5 redirected seawater through six ports (similar to the device described in [7]). Each port was fitted with 25 mm diameter 0.2 µm pore size polysulfone filters (Supor, Pall Gelman, Port Washington, NY, USA). The OCE-5 was programmed to filter one sample every 30 min when sailing over target zones. The tubing and connections of the OCE-5 were thoroughly flushed with 70% EtOH and Milli-Q water between sampling zones. Filtered volumes ranged between 1200 and 2000 ml depending on biomass loading, with filtration times ranging from 10 to 22 min. Filters were stored in bead beater tubes containing a mix of 0.1 mm and 0.5 mm diameter glass beads (Oakton Instruments, Vernon Hills, IL, USA) and immediately stored at -80ºC.

*DNA extractions and quantitative PCR (qPCR)*

DNA was extracted using the DNeasy Plant Mini Kit (Qiagen, Courtaboeuf, France) with additional freeze-thaw bead beating and proteinase K steps before the kit purification and elution to 80 µl in RNase-free water as previously described [8].

The abundance of diazotrophs was determined using TaqMan qPCR assays and previously published primer-probe sets for *Trichodesmium,* UCYN-A1, UCYN-B, UCYN-C and γ-24474A11 (the latter hereafter referred to as Gamma A) targeting the *nifH* gene [8–10]*.* The qPCR was run in 25 µl reactions consisting of 12.25 µl TaqMan PCR Master Mix (Applied Biosystems, Villebon Sur Yvette, France), 1 µl of the forward and reverse primers at 10 µM (HPLC purified, Eurofins, Nantes, France), 0.25 µl probe at 10 µM, 8.25 µl PCR grade water, 0.25 µl bovine serum albumin at 10.08 µg µl^-1^, and 2 µl standard or template sample. The qPCR program was run on a CFX96 Real-Time System thermal cycler (BioRad, Marnes-la-Coquette, France) and consisted of 2 min at 50ºC, 10 min at 95ºC continued by 45 cycles of 15 s at 95ºC and 1 min at 64ºC. The annealing temperature was changed to 60ºC for UCYN-A1 qPCR runs [10]. Standard dilutions (10^7^-10^1^ gene copies) were run in duplicate, and samples and no-template controls (NTCs) in triplicate. NTCs did not show any amplification. The efficiency was 98 - 113%. Inhibition tests were carried out on all samples and each primer-probe set by adding the 2 µl of the 10^5^ copy standard to each sample. No inhibition was observed. The limit of detection and detected but not quantifiable limits were 1 and 8 gene copies per reaction, respectively.

*Nutrients and N_2_ fixation rates*

In zone 3 only, samples were collected manually from the underway outlet for dissolved inorganic nutrient and N_2_ fixation rate analyses. For nutrients, seawater was collected in acid-clean 20 ml polyethylene tubes and stored at -20ºC until analyses. Ashore, samples were analyzed after Aminot and Kérouel [11] on a Bran Luebbe AA3 autoanalyzer. Detection limits were 0.05 µM for both nitrate and phosphate. N_2_ fixation rates were measured from 2.3 l samples collected on transparent polycarbonate bottles and amended with 2 ml 98.9 atom % ^15^N_2_ gas (Euroiso-top, Saint-Aubin, France). Bottles were inverted 30 times and incubated in surface water circulated on-deck incubators for 24 h. Incubations were terminated by filtration onto precombusted 25 mm GF/F filters (GE Healthcare, Little Chalfont, UK) and analyzed on an Integra CN elemental analyzer coupled to an isotope ratio mass spectrometer (Sercon Instruments, Crewe, UK) as previously described [12]. To avoid an underestimation of N_2_ fixation rates due to the incomplete dissolution of ^15^N_2_ gas into seawater samples [13], the ^15^N atom % enrichment of incubated seawater was measured from 12 ml headspace-free samples collected in exetainer tubes (Labco, Ceredigion, UK), stored at 4ºC and analyzed on a membrane inlet mass spectrometer (Pfeiffer, MD, USA) after Kana et al. [14]. The high-resolution sampling approach applied here did not permit sampling in triplicates for N_2_ fixation rate measurements using ^15^N_2_ isotope labeling. Hence, minimum quantifiable rates (MQR) are not reported. Nevertheless, parallel measurements of N_2_ fixation rates at discrete stations during the same cruise (which were labeled with the same volume of ^15^N_2_ gas, equally incubated and analyzed with the same EA-IRMS and MIMS equipment) showed MQR ranging from 0.2 to 0.4 nmol N l^-1^ d^-1^ (S. Bonnet, personal communication).

*Statistical analyses*

Outlier values of diazotroph *nifH* gene copy abundance were removed by the interquartile range method. *nifH* gene copy number data were normally distributed and hence Pearson correlations were used to test their correlation with environmental data (i.e. temperature, salinity and fluorescence) using the Hmisc and corrgram packages in R [15, 16]. Fine-scale structure parameters included FSLE, OW and ADT (see above).

An redundancy analysis (RDA) triplot was computed on Hellinger-transformed *nifH* gene copy abundance data as response variables and fine-scale structure data as explanatory variables using the R package vegan [17]. To avoid misinterpreting zero values of FSLE as absence of data in RDA calculations, FSLE was treated as a non-quantitative variable with two levels: i) FSLE>0.05 d^-1^ indicating the presence of Lyapunov coherent structures (LCS) such as fronts or filaments with an intensity corresponding to the FSLE value, and ii) FSLE<0.05 d^-1^ indicating the absence of significant structures (non-LCS) with a value equal to zero (Table S1). The FSLE value threshold that separates LCS from non-LCS is based on trivial sensitivity tests. The nonlinear dynamics of the ocean imply that the FSLE field depends on the local current circulation and, to the best of our knowledge, a universal threshold value to separate the maxima from the background FSLE values is not available. Fortunately, the exponential nature of the FSLE field implies that the LCS identification is little sensitive to the threshold value, as shown in Fig. 2 where large areas in black (i.e. value much lower than the threshold) are separated by thin lines of values greater than the threshold. The threshold used here is the same applied previously in the same region [18]. OW and ADT were treated as continuous variables. The homogeneity of dispersions within each factor in the RDA was tested using the betadisper function also in vegan [19]. Inorganic nutrient concentrations and N_2_ fixation rates were only measured in zone 3 and thus not included in the RDA. The relation of these parameters with diazotroph abundances in zone 3 was then checked by Spearman rank correlations (Fig. S5).

The significance of interactions between the abundance of each diazotroph group and the fine-scale structure parameters was tested by multivariate analysis of variance (ANOVA) with fine-scale variables defined by the following categories (Table S1; Fig. S1). The OW parameter was categorized as “core” or “edge”, where core regions are under the influence of an eddy (OW<0) and edge regions represent a frontier where shear dominates (OW>0) [20]. FSLE was categorized as LCS or non-LCS as described above (Table S1). ADT was split into three levels (“low”, “medium” and “high”) according to the distribution of data across the three sampled zones (Fig. S1).

**2. Supplementary Tables**

**Table S1:** Categorical classification of the physical parameters Okubo-Weiss (OW), finite size Lyapunov exponents (FSLE), and absolute dynamic topography (ADT) as used for statistical tests.

| Station | Zone | OW (d^-2^) | OW categorical | FSLE (d^-1^) | FSLE categorical | ADT (m) | ADT categorical |
| --- | --- | --- | --- | --- | --- | --- | --- |
| 153 | zone1 | -0.46 | core | 0 | non-LCS | 1.19 | high |
| 154 | zone1 | -0.49 | core | 0 | non-LCS | 1.17 | mid |
| 155 | zone1 | -0.38 | core | 0 | non-LCS | 1.17 | mid |
| 156 | zone1 | -0.12 | core | 0 | non-LCS | 1.17 | mid |
| 157 | zone1 | -0.11 | core | 0 | non-LCS | 1.14 | mid |
| 158 | zone1 | 0.42 | edge | 0 | non-LCS | 1.14 | mid |
| 159 | zone1 | 0.5 | edge | 0.39 | LCS | 1.14 | mid |
| 160 | zone1 | 0.41 | edge | 0.34 | LCS | 1.14 | mid |
| 161 | zone1 | 0.33 | edge | 0 | non-LCS | 1.13 | mid |
| 162 | zone1 | 0.2 | edge | 0.19 | LCS | 1.13 | mid |
| 163 | zone1 | -0.14 | core | 0 | non-LCS | 1.13 | mid |
| 164 | zone1 | -0.2 | core | 0 | non-LCS | 1.13 | mid |
| 165 | zone1 | -0.06 | core | 0 | non-LCS | 1.13 | mid |
| 166 | zone1 | 0.19 | edge | 0 | non-LCS | 1.13 | mid |
| 167 | zone1 | 0.18 | edge | 0 | non-LCS | 1.14 | mid |
| 168 | zone1 | 0.18 | edge | 0 | non-LCS | 1.14 | mid |
| 169 | zone1 | 0.26 | edge | 0.33 | LCS | 1.14 | mid |
| 170 | zone1 | 0.36 | edge | 0.17 | LCS | 1.14 | mid |
| 171 | zone2 | 0.08 | edge | 0 | non-LCS | 1.14 | mid |
| 172 | zone2 | 0.11 | edge | 0 | non-LCS | 1.14 | mid |
| 173 | zone2 | 0.3 | edge | 0 | non-LCS | 1.14 | mid |
| 174 | zone2 | 0.54 | edge | 0.12 | LCS | 1.1 | low |
| 175 | zone2 | 0.09 | edge | 0.39 | LCS | 1.13 | mid |
| 176 | zone2 | -0.31 | core | 0 | non-LCS | 1.1 | low |
| 177 | zone2 | -0.69 | core | 0.18 | LCS | 1.1 | low |
| 178 | zone2 | -0.63 | core | 0.18 | LCS | 1.07 | low |
| 179 | zone2 | -0.17 | core | 0.18 | LCS | 1.06 | low |
| 180 | zone2 | -0.18 | core | 0.17 | LCS | 1.06 | low |
| 181 | zone2 | -0.04 | core | 0 | non-LCS | 1.06 | low |
| 182 | zone2 | -0.22 | core | 0 | non-LCS | 1.08 | low |
| 183 | zone2 | 0.11 | edge | 0 | non-LCS | 1.16 | mid |
| 184 | zone2 | 0.17 | edge | 0.23 | LCS | 1.16 | mid |
| 185 | zone2 | 0.18 | edge | 0.2 | LCS | 1.21 | high |
| 186 | zone2 | -0.09 | core | 0.14 | LCS | 1.25 | high |
| 187 | zone2 | -0.55 | core | 0.13 | LCS | 1.25 | high |
| 188 | zone2 | -0.7 | core | 0 | non-LCS | 1.28 | high |
| 189 | zone2 | -0.05 | core | 0.16 | LCS | 1.27 | high |
| 190 | zone2 | 0.21 | edge | 0.14 | LCS | 1.25 | high |
| 191 | zone2 | 0.4 | edge | 0 | non-LCS | 1.23 | high |
| 192 | zone2 | 0.35 | edge | 0 | non-LCS | 1.23 | high |
| 193 | zone2 | 0.3 | edge | 0.11 | LCS | 1.22 | high |
| 194 | zone2 | 0.26 | edge | 0.14 | LCS | 1.21 | high |
| 195 | zone2 | 0.09 | edge | 0 | non-LCS | 1.21 | high |
| 196 | zone2 | 0.04 | edge | 0 | non-LCS | 1.22 | high |
| 197 | zone2 | -0.08 | core | 0.16 | LCS | 1.22 | high |
| 198 | zone2 | -0.16 | core | 0.2 | LCS | 1.22 | high |
| 199 | zone2 | -0.06 | core | 0.2 | LCS | 1.22 | high |
| 200 | zone2 | -0.01 | core | 0 | non-LCS | 1.22 | high |
| 201 | zone3 | -0.04 | core | 0 | non-LCS | 1.17 | mid |
| 202 | zone3 | -0.66 | core | 0.23 | LCS | 1.17 | mid |
| 203 | zone3 | -0.77 | core | 0 | non-LCS | 1.14 | mid |
| 204 | zone3 | -0.93 | core | 0 | non-LCS | 1.14 | mid |
| 205 | zone3 | -1.1 | core | 0 | non-LCS | 1.14 | mid |
| 206 | zone3 | -1.15 | core | 0 | non-LCS | 1.14 | mid |
| 207 | zone3 | -1.08 | core | 0 | non-LCS | 1.13 | mid |
| 208 | zone3 | -0.82 | core | 0 | non-LCS | 1.13 | mid |
| 209 | zone3 | -0.79 | core | 0 | non-LCS | 1.13 | mid |
| 210 | zone3 | -0.65 | core | 0 | non-LCS | 1.14 | mid |
| 211 | zone3 | 0.08 | edge | 0 | non-LCS | 1.14 | mid |
| 212 | zone3 | 0.16 | edge | 0 | non-LCS | 1.14 | mid |
| 213 | zone3 | 0.25 | edge | 0 | non-LCS | 1.17 | mid |
| 214 | zone3 | 0.79 | edge | 0.19 | LCS | 1.17 | mid |
| 215 | zone3 | 0.86 | edge | 0.43 | LCS | 1.17 | mid |
| 216 | zone3 | 0.82 | edge | 0.19 | LCS | 1.19 | high |
| 217 | zone3 | 0.72 | edge | 0 | non-LCS | 1.19 | high |
| 218 | zone3 | 0.63 | edge | 0 | non-LCS | 1.19 | high |
| 219 | zone3 | 0.56 | edge | 0.27 | LCS | 1.19 | high |
| 220 | zone3 | 0.12 | edge | 0 | non-LCS | 1.18 | high |
| 221 | zone3 | 0.01 | edge | 0 | non-LCS | 1.18 | high |
| 222 | zone3 | -0.07 | core | 0.13 | LCS | 1.18 | high |
| 223 | zone3 | -0.06 | core | 0 | non-LCS | 1.17 | mid |
| 224 | zone3 | 0.21 | edge | 0 | non-LCS | 1.19 | high |
| 225 | zone3 | 0.11 | edge | 0 | non-LCS | 1.19 | high |
| 226 | zone3 | 0.19 | edge | 0.13 | LCS | 1.18 | mid |
| 227 | zone3 | -0.08 | core | 0 | non-LCS | 1.22 | high |
| 228 | zone3 | -0.02 | core | 0 | non-LCS | 1.19 | high |
| 229 | zone3 | -0.01 | core | 0.48 | LCS | 1.15 | mid |
| 230 | zone3 | -0.1 | core | 0 | non-LCS | 1.15 | mid |
| 231 | zone3 | -0.47 | core | 0.17 | LCS | 1.15 | mid |
| 232 | zone3 | -0.65 | core | 0 | non-LCS | 1.12 | low |
| 233 | zone3 | -0.87 | core | 0 | non-LCS | 1.11 | low |
| 234 | zone3 | -0.8 | core | 0 | non-LCS | 1.11 | low |
| 235 | zone3 | -0.68 | core | 0 | non-LCS | 1.11 | low |
| 236 | zone3 | -0.39 | core | 0.12 | LCS | 1.13 | mid |
| 237 | zone3 | -0.35 | core | 0.11 | LCS | 1.15 | mid |
| 238 | zone3 | 0.15 | edge | 0.2 | LCS | 1.15 | mid |
| 239 | zone3 | 0.13 | edge | 0.13 | LCS | 1.17 | mid |
| 240 | zone3 | 0.26 | edge | 0 | non-LCS | 1.17 | mid |
| 241 | zone3 | 0.3 | edge | 0.11 | LCS | 1.17 | mid |
| 242 | zone3 | 0.11 | edge | 0 | non-LCS | 1.16 | mid |
| 243 | zone3 | 0.08 | edge | 0 | non-LCS | 1.16 | mid |
| 244 | zone3 | 0.02 | edge | 0 | non-LCS | 1.16 | mid |
| 245 | zone3 | 0.06 | edge | 0 | non-LCS | 1.15 | mid |
| 246 | zone3 | 0.09 | edge | 0 | non-LCS | 1.15 | mid |
| 247 | zone3 | 0.12 | edge | 0 | non-LCS | 1.15 | mid |
| 248 | zone3 | 0.09 | edge | 0.19 | LCS | 1.13 | mid |
| 249 | zone3 | 0.08 | edge | 0.22 | LCS | 1.16 | mid |
| 250 | zone3 | 0.06 | edge | 0.23 | LCS | 1.16 | mid |
| 251 | zone3 | 0.06 | edge | 0.23 | LCS | 1.16 | mid |
| 252 | zone3 | 0.11 | edge | 0.21 | LCS | 1.16 | mid |
| 253 | zone3 | 0.14 | edge | 0.21 | LCS | 1.16 | mid |
| 254 | zone3 | 0.16 | edge | 0.23 | LCS | 1.16 | mid |
| 255 | zone3 | 0.16 | edge | 0.23 | LCS | 1.17 | mid |
| 256 | zone3 | 0.21 | edge | 0.12 | LCS | 1.17 | mid |
| 257 | zone3 | 0.17 | edge | 0 | non-LCS | 1.19 | high |
| 259 | zone3 | 0.06 | edge | 0 | non-LCS | 1.2 | high |
| 260 | zone3 | 0.03 | edge | 0.11 | LCS | 1.2 | high |
| 261 | zone3 | 0.01 | edge | 0.11 | LCS | 1.22 | high |
| 262 | zone3 | 0.03 | edge | 0 | non-LCS | 1.22 | high |
| 263 | zone3 | 0.08 | edge | 0 | non-LCS | 1.22 | high |
| 264 | zone3 | 0.09 | edge | 0 | non-LCS | 1.24 | high |
| 265 | zone3 | 0.28 | edge | 0 | non-LCS | 1.24 | high |
| 266 | zone3 | 0.24 | edge | 0 | non-LCS | 1.26 | high |
| 267 | zone3 | 0.17 | edge | 0 | non-LCS | 1.24 | high |
| 268 | zone3 | 0.13 | edge | 0 | non-LCS | 1.23 | high |
| 269 | zone3 | 0.01 | edge | 0 | non-LCS | 1.22 | high |
| 270 | zone3 | -0.03 | core | 0 | non-LCS | 1.22 | high |

**Table S2:** The impact of physical parameters on diazotroph abundance based on one-way ANOVA statistical analyses. Significant results (p ≤ 0.05) are depicted with an asterisk.

| Diazotroph group | Physical parameter | F value | p-value |
| --- | --- | --- | --- |
| *Trichodesmium* | OW | 1.691 | 0.196 |
|  | FSLE | 0.005 | 0.944 |
|  | ADT | 11.03 | *4.61 x10^-5^ |
| UCYN-A | OW | 0.68 | 0.998 |
|  | FSLE | 3.731 | *0.050 |
|  | ADT | 2.693 | 0.072 |
| UCYN-B | OW | 1.601 | 0.209 |
|  | FSLE | 0.6 | 0.441 |
|  | ADT | 1.867 | 0.160 |
| UCYN-C | OW | 3.146 | 0.078 |
|  | FSLE | 5.248 | *0.0239 |
|  | ADT | 5.2 | *0.007 |
| Gamma A | OW | 2.709 | 0.103 |
|  | FSLE | 0.452 | 0.503 |
|  | ADT | 10.27 | *7.95 x10^-5^ |

**3. Supplementary Figures**

**Fig. S1:** Histogram of absolute dynamic topography (ADT) values and divisions into low, mid and high levels. The differences between each pair of groups are statistically significant as confirmed by *t*-tests (low vs mid p = 8.69 x 10^-7^, mid vs high p = 2.2 x 10^-16^ and low vs high p = 5.06 x 10^-13^).


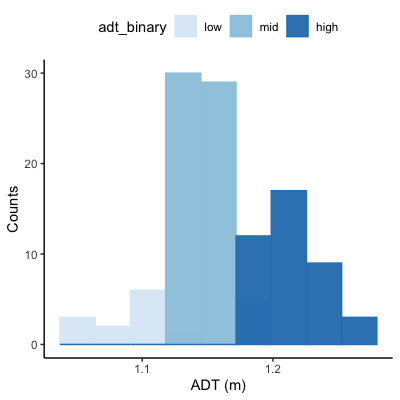


**Fig. S2:** Multivariate redundance analysis (RDA) biplot depicting the variance explained by the fine-scale parameters and qPCR data. The RDA1 and RDA2 axes explained 56.13 and 36.25% of the variance, respectively. The pseudo-F values of each explanatory variable are show in the top left text lines.


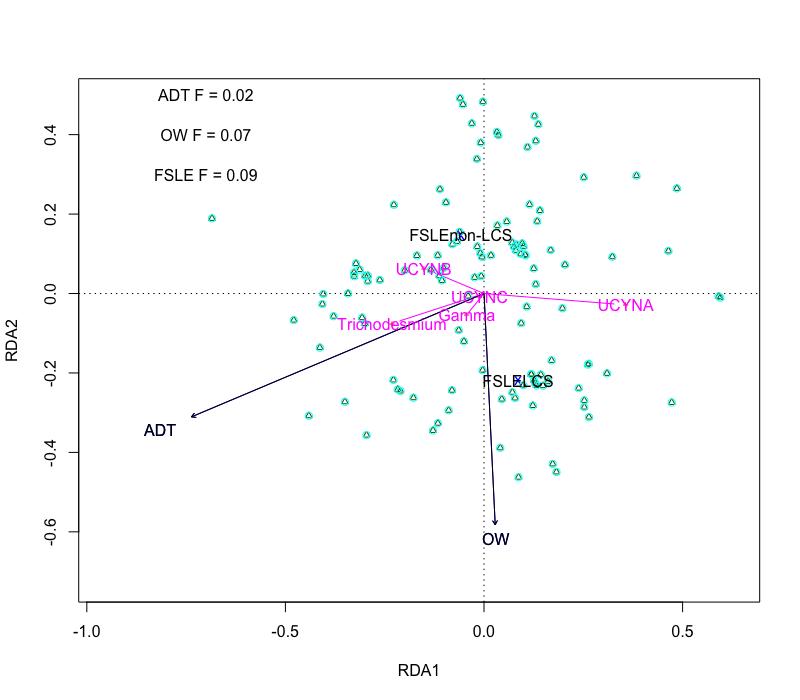


**Fig. S3: Hydrographic parameters from underway measurements in zones 1, 2 and 3.** Temperature, salinity and fluorescence data in zone 1 (a, b, c), zone 2 (d, e, f) and zone 3 (g, h, i). Data were retrieved for each zone on 2^nd^, 4^th^ and 22^nd^ November 2019 for zones 1, 2 and 3, respectively.


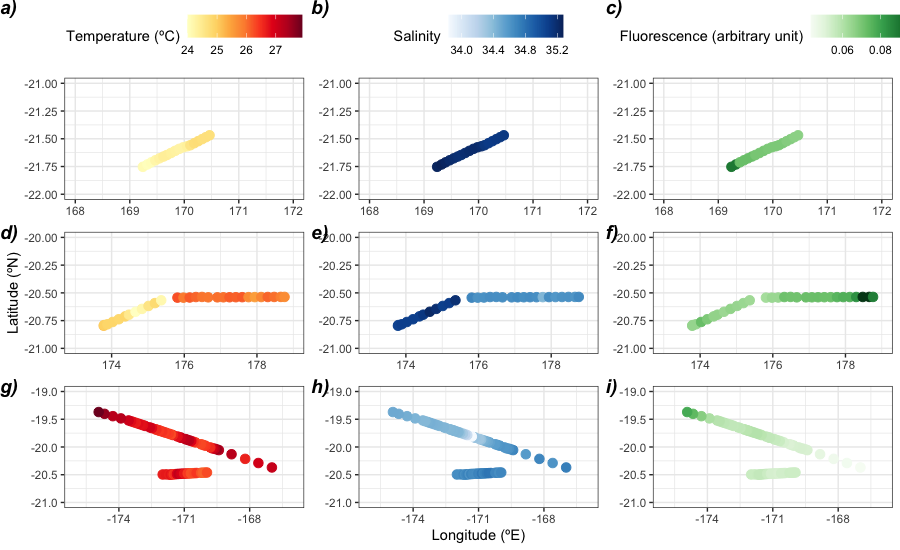


**Fig. S4:** **Pearson correlations between diazotroph abundance and environmental variables from zones 1, 2 and 3.** Biological variables (*nifH* gene counts of *Trichodesmium,* UCYN-A1, UCYN-B, UCYN-C and Gamma per liter of seawater) and environmental variables (temperature, salinity and fluorescence), combining data from zones 1, 2 and 3. Statistically significant (p ≤ 0.05) positive and negative correlations are shown in red and blue circles, respectively. The size of the circles indicates the magnitude of the p-value, while the color tone indicates the Pearson correlation coefficient value as depicted in the color scale. The size of correlation circles of any variable correlated with itself depicts r = 1.


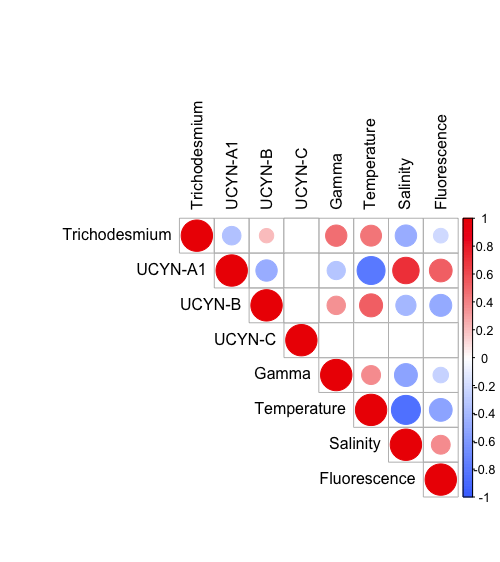


**Fig. S5:** **Pearson correlations between diazotroph abundance, nutrient concentrations and N_2_ fixation rates in zone 3.** Diazotroph abundance (*nifH* gene counts of *Trichodesmium,* UCYN-A1, UCYN-B, UCYN-C and Gamma per liter of seawater), nutrient concentrations (nitrate and phosphate in µM) and N_2_ fixation rates (in nmol N l^-1^ d^-1^) in zone 3. Statistically significant (p ≤ 0.05) positive correlations are shown in red circles. The size of the circles indicates the magnitude of the p-value, while the color tone indicates the Pearson correlation coefficient value as depicted in the color scale. The size of correlation circles of any variable correlated with itself depicts r = 1.


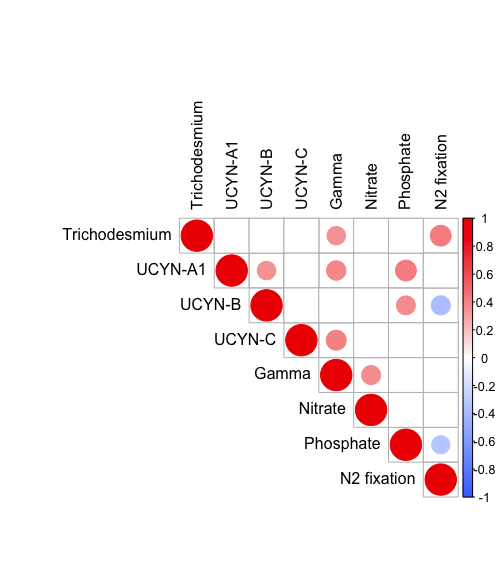


**Fig. S6: Distribution of inorganic nutrients (nitrate and phosphate) and N_2_ fixation rates in zone 3.** Grey dots depict locations where samples for diazotroph abundance are available (see Fig. 1 and Fig. S3), but inorganic nutrient concentration and N_2_ fixation rate data are not available.


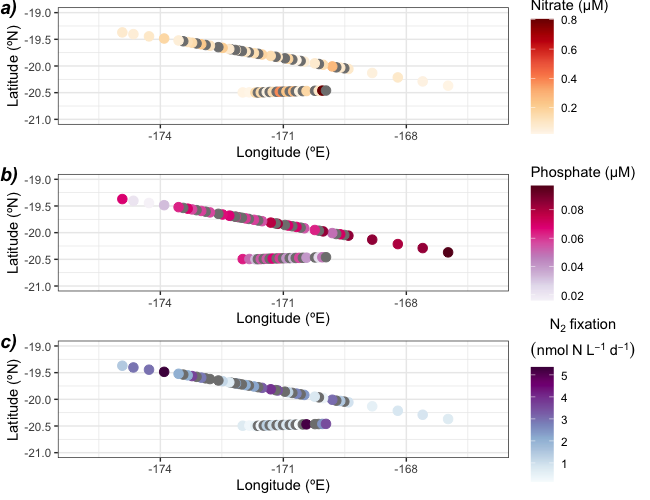


**Fig. S7: Distribution of temperature and inorganic nutrients (nitrate and phosphate) according to fine-scale variables in zone 3.** Boxplots representing the range and mean values of temperature, nitrate and phosphate according to the categorical values of fine-scale variables ADT, FSLE and OW as described in Table S1. The values over horizontal brackets indicate p-values of *t*-tests. Only low versus mid ADT temperature was statistically significant.


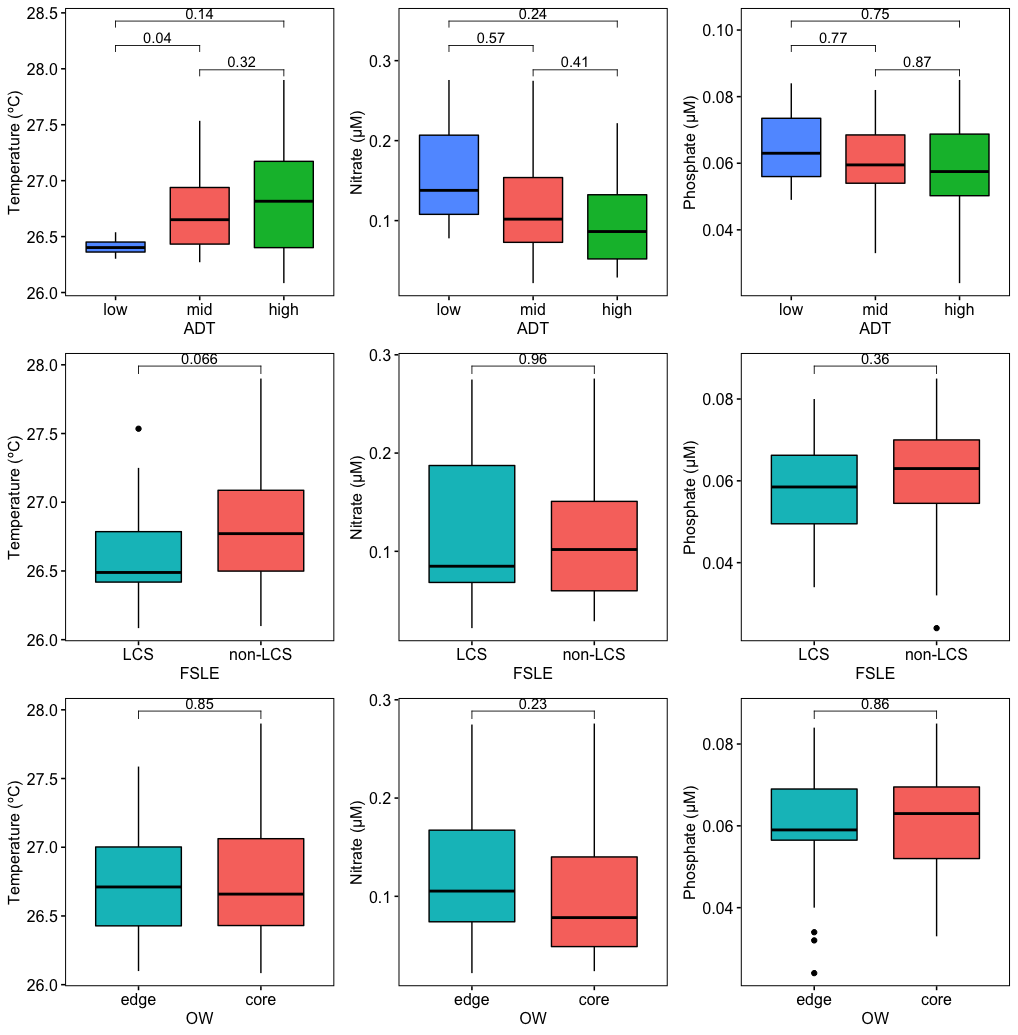


**4. Supplementary References**

1. Doglioli AM et al. A Software package and hardware tools for in situ experiments in a Lagrangian reference frame. *J Atmos Ocean Technol* 2013; **30**: 1940–1950.

2. Petrenko AA et al. A review of the LATEX project: mesoscale to submesoscale processes in a coastal environment. *Ocean Dyn* 2017; **67**: 513–533.

3. D’Ovidio F et al. The biogeochemical structuring role of horizontal stirring: Lagrangian perspectives on iron delivery downstream of the Kerguelen Plateau. *Biogeosciences* 2015; **12**: 5567–5581.

4. Moutin T, Doglioli MA, de Verneil A, Bonnet S. Preface: The Oligotrophy to the UlTra-oligotrophy PACific Experiment (OUTPACE cruise, 18 February to 3 April 2015). *Biogeosciences* 2017.

5. Rousselet L et al. Large- to submesoscale surface circulation and its implications on biogeochemical/biological horizontal distributions during the OUTPACE cruise (Southwest Pacific). *Biogeosciences* 2018.

6. d’Ovidio F, De Monte S, Alvain S, Dandonneau Y, Levy M. Fluid dynamical niches of phytoplankton types. *Proc Natl Acad Sci* 2010.

7. Holser RR, Goni MA, Hales B. Design and application of a semi-automated filtration system to study the distribution of particulate organic carbon in the water column of a coastal upwelling system. *Mar Chem* 2011; **123**: 67–77.

8. Moisander PH, Beinart RA, Voss M, Zehr JP. Diversity and abundance of diazotrophic microorganisms in the South China Sea during intermonsoon. *ISME J* 2008; **2**: 954–967.

9. Church MJ, Jenkins BD, Karl DM, Zehr JP. Vertical distributions of nitrogen-fixing phylotypes at Stn ALOHA in the oligotrohic North Pacific Ocean. *Aquat Microb Ecol* 2005; **38**: 3–14.

10. Thompson A et al. Genetic diversity of the unicellular nitrogen-fixing cyanobacteria UCYN-A and its prymnesiophyte host. *Environ Microbiol* 2014; **16**: 3238–3249.

11. Aminot A, Kérouel R. Dosage automatique des nutriments dans les eaux marines: méthodes en flux continu. 2007. Editions Quae.

12. Bonnet S et al. In-depth characterization of diazotroph activity across the western tropical South Pacific hotspot of N_2_ fixation (OUTPACE cruise). *Biogeosciences* 2018; **15**: 4215–4232.

13. Mohr W, Großkopf T, Wallace DWR, LaRoche J. Methodological underestimation of oceanic nitrogen fixation rates. *PLoS One* 2010; **5**: 1–7.

14. Kana TM et al. Membrane lnlet Mass Spectrometer for Rapid Environmental Water Samples. *Anal Chem* 1994; **66**: 4166–4170.

15. Team Rs. RStudio: Integrated Development for R. *PBC Boston*. .

16. Friendly M. Corrgrams: Exploratory displays for correlatigon matrices. *Am Stat* 2002; **56**: 316–324.

17. Oksanen J. Vegan: ecological diversity. *R Packag Version 24-4* . 2017.

18. Rousselet L, Doglioli AM, Maes C, Blanke B, Petrenko AA. Impacts of mesoscale activity on the water masses and circulation in the Coral Sea. *J Geophys Res Ocean* 2016; **121**: 7277–7289.

19. Anderson MJ. Distance-based tests for homogeneity of multivariate dispersions. *Biometrics* 2006.

20. Elhmaïdi D, Provenzale A, Babiano A. Elementary Topology of Two-Dimensional Turbulence from a Lagrangian Viewpoint and Single-Particle Dispersion. *J Fluid Mech* 1993; **257**: 533–558.
